# Emergency Air Transport of Patients with Acute Chest Pain in the Adriatic Islands of Croatia: A Four-Year Analysis

**DOI:** 10.3390/ijerph20075422

**Published:** 2023-04-06

**Authors:** Antonija Zanic, Vedran Kovacic, Ivana Jukic

**Affiliations:** 1 Institute of Emergency Medicine of Split–Dalmatia County, 21000 Split, Croatia; 2 Internal Medicine Department, Division of Emergency and Intensive Medicine with Clinical Pharmacology and Toxicology, University Hospital of Split, 21000 Split, Croatia; 3 School of Medicine, University of Split, 21000 Split, Croatia; 4 Internal Medicine Department, Gastroenterology Division, University Hospital of Split, 21000 Split, Croatia

**Keywords:** air transport, acute coronary syndrome, helicopter emergency medical service

## Abstract

Background: An efficient first-aid system usually supports ground services with a helicopter emergency medical service (HEMS). An HEMS is important for patients with acute chest pain on remote islands. The current study sought to identify the characteristics of HEMS in acute chest pain cases on the Croatian Adriatic islands over a four-year period. Methods: We conducted a four-year observational study to investigate HEMS from Adriatic islands. The study population consisted of all patients with acute coronary syndrome or pulmonary embolisms who were urgently transferred by HEMS to the University Hospital in Split 1 June 2018–1 June 2022. Results: During the observation period, 222 adult patients (67 females, or 30.2%) were urgently transferred. The mean age was 71.81 ± 13.42 years. The most common diagnosis was ST-elevated myocardial infarction (113, 50.9%). Most of the HEMS cases were from Hvar (91, 41.0%). The mean call-to-flight time was 19.10 ± 10.94 min, and the total time from call to hospital was 68.50 ± 22.29 min. The total time from call to hospital was significantly correlated with call-to-flight time (r = 0.761, *P* < 0.001). Of the 222 participants, 5 (2.25%) were transported for more than 120 min, and 35 (15.8%) were transported for more than 90 min. Conclusion: This study provided a detailed insight into HEMS in the area of the Croatian Adriatic islands. The average time from the call to the helicopter taking off was 19.10 min. An increase in dispatching time has a significant impact on the prolongation of the total time for the hospital admission. Shortening the response time is critical to reducing hospital arrival time.

## 1. Introduction

Efficient first-aid systems perform the complementary action of all intervention methods and it is becoming a usual practice in national intervention systems to support ground services with air assets, these often being medical helicopters. Of all ambulance transfers, the helicopter emergency medical service (HEMS) makes about 3% [1].

HEMS is generally accepted to have a shorter prehospital time than ground-based medical transportation, information which can be used to save patients by timing their arrival at the hospital within the “golden hour” [2]. Consequently, HEMS has a substantial influence on saving lives when used in rural or island settings. However, it can also have important advantages in densely populated metropolitan areas [3].

In acute coronary syndrome, timely intervention is extremely important, and the phrase “time is muscle” emphasizes the importance of time in the treatment of these patients [4]. Studies have found a significant relationship between long-term ischemic periods and poor results [5]. Recent guidelines suggest a less than 60 min target from taking the ECG (electrocardiogram) to intervention in cases of acute myocardial infarction with ST segment elevation [6]. Bearing such evidence and guidelines in mind, the use HEMS is especially important for patients with acute chest pain in rural areas or in areas where land traffic is poor, such as remote islands [7].

The Republic of Croatia is a Mediterranean country that occupies an area of 56,542 square kilometres with a coastline of 5835 km and more than 1000 islands. Helicopter transport is the fastest and, in some distant places, the only practical method of transporting patients for the majority of Croatia’s Adriatic coast and islands. Split–Dalmatia County, with almost half a million inhabitants, is the largest county in the Republic of Croatia, with a total area of 14,405 km^2^, and includes eight inhabited islands. Dubrovnik–Neretva County is the southernmost Croatian county with about 130,000 inhabitants and covers an area of 9272 km^2^ with six inhabited islands [8].

Most of the patients in Croatia are currently transported by vehicles for emergency medical care. Military helicopters are employed to support the nation’s emergency medical assistance system. When the weather permits, some patients are transferred by boat, rather than by helicopter, from the Adriatic islands to the city of Split. Military helicopters used for HEMS necessitate certain take-off and landing circumstances. There is often a location on every larger island where helicopters can land in case of emergency. The 93rd Airbase Zemunik in Zadar commands the squadron of transport helicopters stationed in Split–Dalmatia County at the Split–Divulje military air base. The MI-8T, MI-8MTV, and MI-17MTV military helicopters are available for use. Dedicated clinical staff members, including an urgent care physician and a certified specialized nurse, regularly provide care for HEMS patients [9].

A special challenge for emergency services is the transportation of seriously ill patients who are located on remote islands. It has been demonstrated that helicopter air transport is the best solution for the medical transportation of critically ill or injured people who require the quickest possible transportation from an island to the hospital [10]. Acute helicopter transport is essential for both traumatic and non-traumatic critically ill patients. There is a very significant proportion of non-traumatic acute cardiovascular and pulmonary patients who need emergency air transportation to the hospital. In one study, up to 25% of these patients were of this kind [11]. Even the most severe SARS-CoV-2-pneumonia patients with respiratory insufficiency may be effectively transferred from very isolated ocean islands, despite the fact that many hours of helicopter transport expose patients to considerable hypoxemia [12].

Given the size, complexity, and self-organization of the air transportation system, it is crucial to strictly regulate it using an air route optimization approach in order to minimize traffic flows [13]. At the same time, it is necessary to take into account the rational transportation of patients because it is well known from the literature that air transport is subject to irrational use and the insufficient utilization of particular take-off and landing units [14]. Rational utilization of the multi-airport networks during air transportation is a challenge and is subject to large variations in the amount of air transport available [15].

Transportation by any water surface, be it by sea or river, is always difficult, challenging, and consumes enormous resources [16]. Therefore, the simplest way of providing emergency transportation from the islands to the mainland is by helicopter. Data on HEMS for the Adriatic region of Croatia are still lacking. However, given its touristic potential, information on emergency air transportation from these islands to the hospital is of the utmost significance.

The current study sought to evaluate the efficacy and characteristics of HEMS in cases of acute chest pain on Croatian islands in two Adriatic counties over a four-year period. The main objectives of the study were to show the time of the HEMS response and to determine the differences between the times of transport to the hospital from distant islands. The article presents the demographic, clinical, and technical aspects of HEMS from remote islands to a hospital. The duration of helicopter transport in all phases of the flight, as well as the duration of transport from all remote island stations, is shown in detail.

### Organization of the Manuscript

There are five key sections of the article. The first is the introduction, with a brief literature review regarding emergency air transport problems. A thorough explanation of the research technique and methodology is provided in the second section of the article. The third section presents the results of the research. The discussion is contained in the fourth section. The article ends with conclusions.

## 2. Materials and Methods

A four-year observational retrospective cross-sectional study was conducted to investigate HEMS from the Adriatic islands at the University Hospital of Split in Croatia. The hospital, as the largest hospital centre on the eastern Adriatic coast and the central health institution in the entire southern region of Croatia, is a regional referral centre for percutaneous coronary interventions. Our 1500-bed hospital, with more than 50,000 patients hospitalized per year, serves about a million citizens of the Republic of Croatia, approximately 500,000 residents of the southern part of Bosnia and Herzegovina, and approximately 500,000 tourists during the summer season.

The study protocol was approved by the Ethics Committee at the Institute of Emergency Medicine of Split–Dalmatia County (Reference Number: 2181-148-01-09-22-0002). The study was conducted according to the principles of the Declaration of Helsinki.

### 2.1. Study Population

The study population consisted of all patients with a presumed diagnosis of acute coronary syndrome or pulmonary embolism who were transferred from Adriatic inhabited islands to the University Hospital in Split during a four-year period running from 1 June 2018 to 1 June 2022. The study included patients over 18 years who were urgently transported by HEMS from the Adriatic islands to the hospital due to acute chest pain or discomfort and immediately admitted to the University Hospital in Split. Exclusion criteria were urgent HEMS for other life-threatening diagnoses. Eligible patients in the outpatient emergency facilities on the islands were examined and initially treated by the physicians on duty. A presumed diagnosis was established depending on the clinical presentation, exam, ECG, qualitative troponin test, and severity of symptoms. Prior to hospitalization, all participants (100%) had their initial ECG performed at a regional outpatient station. Subsequently, the HEMS decision was made, the responsible physician called the central helicopter dispatch team at the air base for Divulje–Split, and the urgent air transport sequence was initiated. All of the HEMS flights took off from the military air base Split–Divulje, which is located on the mainland near Split. All patients transported by helicopter were equipped with all instruments, drugs, oxygen, and monitors necessary to monitor and maintain vital functions, including a defibrillator, with the ability to monitor heart rate through 12 channels of ECG, non-invasive heart pressure, and oxygen saturation. A physician with experience in emergency care and a nurse with specialized training were present with the patient during the flight.

Data collected from electronic records included information in regard to demographics, disease conditions, detailed transportation courses and destinations, the name of the outpatient hospital island facility, dates, dispatch times, flight times, and hospital arrival times. The call-to-flight time was defined as the time from the first call directed to the HEMS dispatch centre until the helicopter took off from Split–Divulje military air base. A first call to the HEMS dispatch centre was made by physicians on duty in the outpatient emergency facility on the particular island. The flight-to-heliport time was defined as the time that it took for the helicopter to take-off from an air base, pick up the patient from an island, and land at the University Hospital Split heliport. The heliport-to-hospital time was defined as the time that it took from the patient landing until admission to one of the hospital departments. The total time from call to hospital was defined as the total time between the first call directed to the air base and admission to the hospital.

### 2.2. Statistical Analysis

Descriptive statistical calculations and data were presented as a number (percentage) for qualitative variables (number of participants in groups). Quantitative variables were expressed as the arithmetic mean ± standard deviation (age of participants, duration of flights). Qualitative data between groups were compared with a chi-square (χ^2^ = ∑(O_i_ − E_i_)^2^/E_i,_ where O_i_ = observed value (actual value) and E_i_ = expected value) or Fisher’s exact tests [P = (a + b)! (c + d)! (a + c)! (b + d)!/(a!b!c!d!n!)], as appropriate (differences in number of flights from different counties after and before the COVID-19 pandemic, differences in number of flights from different counties transported lasting for more or less than 90 min, and more or less than 60 min). A one-sample chi-square method was used to test differences between the numbers of flights from different islands. Differences between two groups of quantitative data were compared using the unpaired Student’s *t* test ($t=¯\mathrm{yA}-¯\mathrm{yB}/\surd s^{2}p/\mathrm{nA}+s^{2}p/\mathrm{nB}$; *t* statistic has nA + nB − 2 degrees of freedom, Sp is the pooled standard deviation of the sample, s^2^p is the pooled variance of the sample) for gender differences and differences between durations of flights before and after COVID-19 pandemics. Differences in durations of flights between seven islands’ outpatient stations were tested using the one-way ANOVA test. ANOVA F were calculated as F = SSB/SSW; SSB (mean sum of squares between the group) = sum of squares between the group SSb/DFb; DFb = Degree of freedom = K − 1 where K is the number of group, and SSb = ΣNi(Xi − Xt)^2^ where *Xi* is mean of group i and Xt is mean of all the observations; SSW = sum of squares within the group SSW/DFw; DFw = degrees of freedom = N − K where K is the number of group, and N is total number of observations in all the group; SSW = Σ(Xij − Xj)^2^ where Xij is the observation of each group j.

The correlations between the quantitative data were calculated using Pearson’s correlation coefficient calculated as:

$$r=\frac{n\left(\Sigma \mathrm{xy}\right)-\left(\Sigma x\right)\left(\Sigma y\right)}{\sqrt{\left[n\Sigma x^{2}-\left(\Sigma x^{2}\right)]\;[n\Sigma y^{2}-\left(\Sigma y^{2}\right)\right]}}$$

where r = Pearson coefficient; n = number of pairs of the stock; ∑xy = sum of products of the paired stocks; ∑x = sum of the x-scores; ∑y = sum of the y scores; ∑x^2^ = sum of the squared *x*-scores; ∑y^2^ = sum of the squared y scores. Pearson’s correlation coefficient was used to test for correlations between various flight duration times and age of participants. A regression plot with the equation of linear regression was constructed to demonstrate the correlation between the total time from call-to-hospital and call-to-flight time. Equation of linear regression was expressed as Y = a + bX, where Y is the dependent variable (y axis), x is the independent variable (x axis), b is the slope of the line, and a is the y-intercept. Statistical analysis was performed with SPSS software for Windows (IBM SPSS Statistics for Windows, version 26.0, Armonk, NY, USA). *P* values of less than 0.05 were considered significant.

## 3. Results

**Figure 1:**
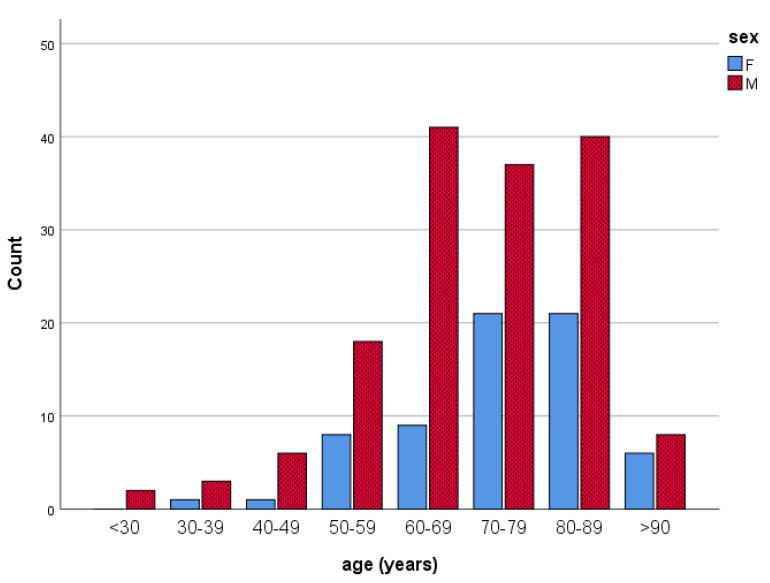
Gender and age distribution of the participants (n = 222).

During the four-year observation period, 222 adult patients (67 females or 30.2%, 155 males or 69.8%) who presented in outpatient emergency services with chest pain were urgently transferred from the Adriatic islands to the hospital admission at the University Hospital in Split, Croatia. The average age of the participants was 71.81 ± 13.42 years (range 25–98). (Figure 1) Men presented significantly more frequently with the dieasethan women in all age groups. We also detected, during the four years, 7 patients with chest pain (3.06% of all patients with chest pain) who could not be transferred by helicopter to the hospital. The reason for the helicopter’s inability to take off was bad weather conditions; in most cases, the issue was a strong wind. These patients were transported by sea (by speedboats or regular shipping lines).

**Figure 2:**
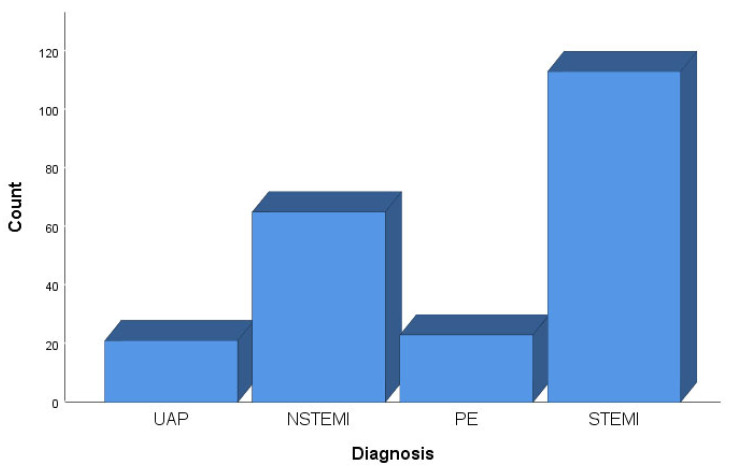
Distribution of diagnosis among subjects (n = 222). Legend: UAP: unstable angina pectoris, NSTEMI: non-ST-elevated myocardial infarction, PE: pulmonary embolism, STEMI: ST-elevated myocardial infarction.

The presumed diagnoses were unstable angina pectoris (21 or 9.5%), non-ST-elevated myocardial infarction (65 or 29.3%), pulmonary embolism (23 or 10.4%), and ST-elevated myocardial infarction (113 or 50.9%). (Figure 2) ST-elevated myocardial infarction was the most common presumptive diagnosis for which emergency helicopter transport was requested to the hospital. Only 29 participants (13.1%) had a positive qualitative troponin test result. There were no significant differences in transportation times between the diagnoses.

Table 1 shows the times of helicopter medical service, including the total time from call to the hospital. In total, 155 h and 40 min of helicopter flight (in the air) were achieved. The mean total time from call to hospital was 68.50 ± 22.29 min.

The gender differences are demonstrated in Table 2. Females were statistically significantly older than males. Except for the heliport-to-hospital time, males had significantly shorter emergency helicopter transport times.

Flights from seven Adriatic islands were analyzed: Brac (18 participants, 8.1%), Hvar (91 participants, 41.0%), Korcula (88 participants, 39.6%), Solta (13 participants, 5.9%), Lastovo (3 participants, 1.4%), Mljet (2 participants, 0.9%), and Vis (7 participants, 3.2%). (one-sample chi-square = 300.79, *P* < 0.001) The differences in transportation times between islands are demonstrated in Table 3. All helicopter air transport times varied significantly between individual island outpatient units. There was no difference except in the heliport-to-hospital time, which is not in fact the flight time of the helicopter.

The differences in transportation times after and before the COVID-19 pandemic are shown in Table 4. Flight-to-heliport time was significantly shorter after the COVID-19 pandemic.

The differences in the distribution of the number of flights from different countries after the COVID-19 pandemic and before the COVID-19 pandemic are shown in Table 5. (chi square = 5.83, *P* = 0.011). It has been demonstrated that there was a statistically significant drop in flights from Dubrovnik–Neretva County following the COVID-19 epidemic.

**Figure 3:**
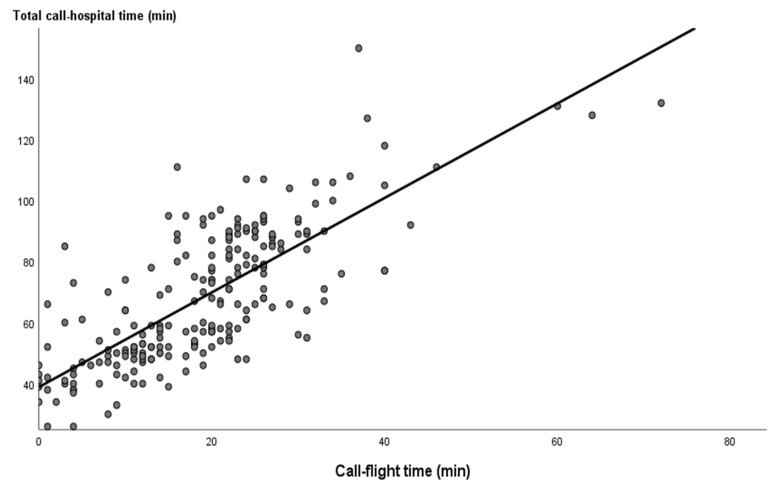
A regression plot with the equation of linear regression demonstrated a correlation between the total time from call to hospital and call to flight time, y = 1.55X + 38.89, r = 0.761, *P* < 0.001.

The total time taken from call to hospital was significantly correlated with age (r = 0.113, *P* = 0.046), call-to-flight time (r = 0.761, *P* < 0.001), flight-to-heliport time (r = 0.113, *P* < 0.001), and heliport-to-hospital time (r = 0.176, *P* = 0.004). The high positive correlation (r = 0.761) between the total time from call to hospital and call-to-flight time is demonstrated as a linear regression plot in Figure 3.

Of the 222 participants, only 5 (2.25%) patients were transported (from call to hospital) for more than 120 min, and 35 (15.8%) patients were transported for more than 90 min. Only one patient from Split–Dalmatia County (0.8%) was transported for more than 90 min; on the contrary, from Dubrovnik–Neretva County, 34 patients (36.56%) were transported for more than 90 min (chi-square = 52.11, *P* < 0.001).

Of the 222 participants, 102 (45.9%) arrived at the hospital in less than 60 min.

Only 2 (2.2%) patients from Dubrovnik–Neretva County were transported in less than 60 min, and 100 (77.5%) patients from Split–Dalmatia County reached the hospital in less than 60 min (chi-square = 123.60, *P* < 0.001).

## 4. Discussion

This four-year observational study provides detailed insight into the availability, efficacy, demographics, and clinical characteristics of urgent air transportation for acute chest pain from the Adriatic islands to the regional area hospital centre of Split–Dalmatia County.

The significance of this study is particularly important since our hospital provides care for the region, which accounts for 1.5 million residents as well as half a million tourists throughout the summer.

Two types of transportation are often available to urgent patients: ground ambulances and helicopters. The pathways they take, as well as the amount of time they spend in transit, vary significantly. Additionally, it has been proven that the transportation of patients via air by helicopter is safer in terms of fewer crashes than transport by ambulance on the ground [17].

The situation is more complicated on remote islands, where it is necessary to combine ground transport and sea transport (usually speedboat). HEMS is typically regarded as a better mode of transportation because of its reduced travel time and low risk of unexpected delays. One study demonstrated that HEMS transport saved 56 min when compared to ground medical transport services [18].

Our research found that the HEMS in this area is well-organized, with a mean time of 68.50 min between the initial call and arrival at the hospital. Almost half (45.9%) of the participants were admitted to the hospital within the “golden hour.” In addition, this study revealed that HEMS is readily available when urgent transport from the island to the hospital is necessary. Namely, only a very small number of cases made helicopter transfer impossible due to weather conditions (only occurring for 3.06 percent of patients with chest discomfort).

However, there are significant disparities in the duration of the transport, which depends on the distance between the islands and the hospital. We demonstrated that HEMS flights from southward islands (Korcula, Lastovo, and Mljet) in Dubrovnik–Neretva County needed significantly more time to reach the hospital than flights from islands in Split–Dalmatia County. From Mljet Island, the mean call-hospital time was 119 min, which substantially exceeded the “golden hour.” However, 98% of patients reached the hospital within an interval of 120 min, which is still acceptable for patients with acute coronary syndrome and pulmonary embolism. Since outcomes depend on timely interventions, the significant difference in transportation times may have an impact on patients with acute coronary syndrome. Time is often used as a substitute for quality measures and is invariably associated with better patient outcomes. In a recent study in Turkey, 39.5% of all analyzed HEMS flights were due to cardiovascular emergencies, with acute myocardial infarction being the most common cause of all cardiovascular emergencies (79.5%) [19]. The study reported a mean flight time of 35.5 ± 23.3 min, but the mean operation transport time was 150.6 ± 279.3 min. Similar to this, many other reports revealed that the most prevalent diagnosis category among patients transported by helicopter ambulances was cardiac emergencies [20,21].

Data reported in the HEARTS study, which included 257 HEMS at patients with STEMI, demonstrated that 67.7% of subjects had an overall transport time (from dispatch to receiving cardiac unit arrival) within 90 min and 91.1% had one within 120 min, with an estimated 1.34 lives saved per 100 HEMS [22].

For instance, the official target proclaimed in Norway is to reach 90% of the population within a time window of 45 min, which includes up to a 15 min reaction time from alert to take-off; however, in practice, there are considerable differences between air bases, conditioned by their geographical distribution [23].

The mean age (71.81 years) and gender ratio (69.8% males) of the patients who participated in our study were shown to be consistent with some previous reports [24]. The total time from call-to-hospital in our cohort was significantly shorter for males, including call-to-flight time. We hypothesized that in males, clinical presentations were more serious and that, as a result, and they were dispatched for and transported sooner. Males were found to be younger than females (mean, 70.54 vs. 74.75 years).

Many healthcare systems in the industrialized world use HEMS as a vital component [25]. HEMS has a huge impact on saving lives in many types of emergencies, and even has uses in urban areas. A helicopter transport service has been used successfully to improve outcomes in emergency perinatal patients [26], in acute ischemic stroke patients (reduced disability after an ischemic stroke) [27], in traumatic patients, especially in a rural area [28], in paediatric emergencies [29], in patients with septic shock [30], in snake envenomation [31], and in patients with myocardial infarction [32]. Even critically ill COVID-19-positive patients can be successfully transferred by HEMS. According to a study from the Netherlands, it is possible to transfer ventilated COVID-19-critical patients by helicopter while minimizing the risk of infection through the correct use of protective equipment by HEMS staff [33].

One of the primary goals of HEMS is to improve the outcomes for patients. A study of patients with STEMI reported a time-based mortality effectiveness of HEMS of approximately 1.2 lives saved per 100 flights [34]. Schoos et al. [32] reported a group of STEMI patients from a distant island and demonstrated that air transport was practical and safe. Furthermore, their 30-day mortality following coronary intervention was equivalent to that of the general population on the mainland.

Taking into account the aforementioned benefits of HEMS, the Republic of Croatia included HEMS in the public health service while delivering emergency medical care. The Republic of Croatia is a full member of both the Joint Aviation Authority (JAA) and the European Aviation Safety Agency (EASA) and has agreed to follow all of the aforementioned organizations’ rules. Joint Aviation Regulations Operations 3 criteria for helicopters are also part of these regulations [35]. These rules specify the requirements helicopters must meet in order to provide helicopter emergency medical service.

Military helicopters are employed in Croatia to support the nation’s emergency medical assistance system. Helicopters are used most often in Split–Dalmatia County for transporting patients from the Adriatic islands to the University Hospital of Split [36].

However, in terms of safety and technological equipment, these helicopters do not satisfy the unique requirements for HEMS. Additionally, there are several drawbacks to using helicopters for emergency aid in Croatia, including the fact that no one organization is in charge of the entire system. The medical personnel who accompany the patient or injured person have not always received the proper specialized training, military helicopters are not designed for emergency aid and, finally, deviations from the standard operating procedures for calling a helicopter have been reported.

In this study, we reported an important drawback: that the dispatching process takes an unacceptable amount of time. Specifically, the mean time from the first call to the beginning of the helicopter taking off was unacceptably long (mean 19.10 ± 10.94, with a maximum of 72 min). Additionally, our study showed that the increase in dispatching time has a significant impact on the extension of the flight-in-air time and, consequently, has a significant impact on the prolongation of the total time frame from the call to admission to the hospital. According to Folkestad et al. [37], the typical HEMS reaction time in a Norwegian environment was around 3 min. Given the positive impact of prompt arrival on the scene, many European health systems demand noticeably reduced response times for HEMS [38]. However, only several studies have shown a clear connection between response time and its impact on morbidity and death [39].

Generally, the duration of the transfer is greatly affected by the distance from the patient’s location to the hospital to which they are to be transported. Since the distance is an unchangeable variable, the acceleration of call-to-take-off time is a crucial factor that significantly affects the total HEMS transportation time. This has been identified as a barrier to improving HEMS operations in Croatia. Long dispatching times could be attributed to the procedural complexities of issuing the necessary bureaucratic permits for helicopter take-offs. Many factors must be met in order to justify a helicopter being dispatched on a rescue operation. As it is a costly operation, it is important to determine the relevant elements of the decision which eliminate the possibility of using alternative means of transportation, that is, to judge whether the helicopter is the only means of transportation that can be operated quickly in rescue operations. In order to speed up the HEMS response time to events and improve the care of the patients being transported by HEMS, it is very important to educate the medical personnel involved in this form of medical transport. The training of on-call personnel stationed at an HEMS airbase is particularly important. It has been demonstrated that such education is simple and inexpensive to achieve through the simulation in situ training [40]. Unfortunately, in the Republic of Croatia, the education of doctors and other personnel related to HEMS is not sufficiently regulated. A small segment of the education of HEMS team members began to be implemented (for both medical and non-medical personnel) in 2015 through a pilot project of the Ministry of Health of the Republic of Croatia.

Finally, we also found that flight-to-heliport transportation time (time of operation in the air) after the COVID-19 pandemic became significantly shorter. The number of HEMS flights increased slightly after the COVID-19 pandemic. Additionally, following the COVID-19 pandemic, the pattern changed, with an increment in the proportion of islands from Split–Dalmatia County in HEMS cases. A similar study in the UK (East of England) demonstrated changing trends after the COVID-19 pandemic: HEMS activations decreased by 24.2% after the COVID-19 pandemic, with an increase of 11.0% in the number of deliberate self-harm incidents [41]. In an additional study conducted in the UK, HEMS activations for out-of-hospital cardiac arrests showed substantial variation (33.6% during COVID-19 compared with 25.8% during the control period the year before) [42].

The present study has some limitations, mainly due to its retrospective observational design and the data being from a single HEMS centre and a single hospital. The study did not include cost and patient safety indicators, which are important in assessing helicopter transport success. Furthermore, additional clinical data on the outcomes for participants are missing.

## 5. Conclusions

The innovation of this research is the inclusion of detailed data on the emergency helicopter transport of patients with chest pain from the Croatian islands to the regional hospital in Split. Such information was not previously available. According to the four-year analysis, STEMI was the most common subgroup of transferred patients with urgent helicopter transport from the Adriatic islands to the regional hospital centre in Split due to acute chest pain. There was a significant difference in the number of HEMS cases between Adriatic islands; HEMS cases were most frequent on the island of Hvar. In 120 min, 98% of participants were transported, while 46% of participants were transported in 60 min. The findings confirm the existence of a relatively well-organized HEMS in this region and identify the reaction time from alert to take-off as a barrier to the better functioning of the HEMS system. Shortening the activation time is essential to reducing the total time between the start of a clinical event and the patient’s arrival at the hospital. The upgrade of HEMS should include an improved management system with clear standard operating procedures. Military helicopters are large, have limited landing options, necessitating the building of a heliport, and should be replaced with smaller transportation helicopters. More multicentric studies with clinical outcomes are needed to clarify the aforementioned conclusions.

## Figures and Tables

**Table 1:** Times from call to hospital admission (n = 222).

	Mean±	Std. Deviation	Minimum	Maximum
Call-to-flight time (min)	19.10±	10.94	0.00	72.00
Flight-to-heliport time (min)	42.08±	15.11	17.00	88.00
Heliport-to-hospital time (min)	7.32±	5.11	0.00	64.00
Total time from call to hospital (min)	68.50±	22.29	26.00	150.00

**Table 2:** Differences between females (n = 67) and males (n = 155) in age and transportation times; Student’s *t* test for independent samples, one-tailed.

	Females		Males		
	Mean±	Std. Deviation	Mean±	Std. Deviation	*P*
Age (years)	74.75±	12.32	70.54±	13.71	0.016 *
Call-to-flight time (min)	21.54±	11.65	18.05±	10.48	0.014 *
Flight-to-heliport time (min)	45.78±	14.76	40.48±	15.02	0.008 *
Heliport-to-hospital time (min)	6.96±	4.14	7.48±	5.48	0.243
Total time from call to hospital (min)	74.27±	23.82	66.01±	21.18	0.005 *

Legend: *: *P* < 0.005.

**Table 3:** Differences in transportation times between islands, one-way ANOVA.

		Mean±	Std. Deviation	F	*P*
Call-to-flight time (min)	Brac	11.83±	7.96	10.256	<0.001 *
	Hvar	14.82±	8.78		
	Korcula	24.99±	11.03		
	Lastovo	22.67±	6.51		
	Mljet	27.00±	15.56		
	Solta	18.69±	11.00		
	Vis	16.43±	5.38		
Flight-to-heliport time (min)	Brac	30.44±	6.24	114.386	<0.001 *
	Hvar	32.58±	5.71		
	Korcula	55.49±	8.99		
	Lastovo	64.33±	1.53		
	Mljet	87.00±	1.41		
	Solta	20.00±	3.37		
	Vis	45.43±	14.48		
Heliport-to-hospital time (min)	Brac	6.50±	3.59	1.675	0.128
	Hvar	6.93±	2.56		
	Korcula	7.42±	4.28		
	Lastovo	7.00±	2.00		
	Mljet	5.00±	2.83		
	Solta	11.38±	15.99		
	Vis	6.43±	2.37		
Total time from call to hospital (min)	Brac	48.78±	8.88	62.646	<0.001 *
	Hvar	54.34±	11.00		
	Korcula	87.90±	16.13		
	Lastovo	94.00±	8.89		
	Mljet	119.00±	11.31		
	Solta	50.08±	16.25		
	Vis	68.29±	16.41		

Legend: *: *P* < 0.005.

**Table 4:** Differences in transportation times before and after the COVID-19 pandemic, Student’s *t* test for independent samples, one-tailed.

	Before COVID-19 Pandemic (n = 103)		After COVID-19 Pandemic (n = 119)		
	Mean±	Std. Deviation	Mean±	Std. Deviation	*P*
Call-to-flight time (min)	18.85±	11.23	19.32±	10.71	0.376
Flight-to-heliport time (min)	44.16±	15.13	40.28±	14.91	0.028 *
Heliport-to-hospital time (min)	7.48±	6.77	7.18±	3.05	0.337
Total time from call to hospital (min)	70.49±	22.47	66.78±	22.08	0.109

Legend: *: *P* < 0.005.

**Table 5:** Distribution of the number of flights from different counties after and before the COVID-19 pandemic. Data are presented as number (percent), chi-square = 5.83, *P* = 0.011.

	Dubrovnik–Neretva County		Split–Dalmatia County	
Before COVID-19 pandemic	52	(55.91%)	51	(39.53%)
After COVID-19 pandemic	41	(44.09%)	78	(60.47%)
Total	93	(100.00%)	129	(100.00%)

## Data Availability

The data presented in this study are available on request from the corresponding author.
